# Dendrimer-Coated Gold Nanoparticles for Efficient Folate-Targeted mRNA Delivery In Vitro

**DOI:** 10.3390/pharmaceutics13060900

**Published:** 2021-06-17

**Authors:** Londiwe Simphiwe Mbatha, Fiona Maiyo, Aliscia Daniels, Moganavelli Singh

**Affiliations:** Nano-Gene and Drug Delivery Group, Discipline of Biochemistry, School of Life Sciences, University of KwaZulu-Natal, Private Bag X54001, Durban 4000, South Africa; londiwem3@dut.ac.za (L.S.M.); fcmaiyo@kabarak.ac.ke (F.M.); DanielsA@ukzn.ac.za (A.D.)

**Keywords:** gold nanoparticles, PAMAM dendrimers, folic acid, mRNA, gene expression

## Abstract

Messenger RNA (mRNA) is not an attractive candidate for gene therapy due to its instability and has therefore received little attention. Recent studies show the advantage of mRNA over DNA, especially in cancer immunotherapy and vaccine development. This study aimed to formulate folic-acid-(FA)-modified, poly-amidoamine-generation-5 (PAMAM G5D)-grafted gold nanoparticles (AuNPs) and to evaluate their cytotoxicity and transgene expression using the luciferase reporter gene (F*Luc*-mRNA) in vitro. Nanocomplexes were spherical and of favorable size. Nanocomplexes at optimum nanoparticle:mRNA (*w*/*w*) binding ratios showed good protection of the bound mRNA against nucleases and were well tolerated in all cell lines. Transgene expression was significantly (*p* < 0.0001) higher with FA-targeted, dendrimer-grafted AuNPs (Au:G5D:FA) in FA receptors overexpressing MCF-7 and KB cells compared to the G5D and G5D:FA NPs, decreasing significantly (*p* < 0.01) in the presence of excess competing FA ligand, which confirmed nanocomplex uptake via receptor mediation. Overall, transgene expression of the Au:G5D and Au:G5D:FA nanocomplexes exceeded that of G5D and G5D:FA nanocomplexes, indicating the pivotal role played by the inclusion of the AuNP delivery system. The favorable properties imparted by the AuNPs potentiated an increased level of luciferase gene expression.

## 1. Introduction

Over the years, non-viral gene delivery modalities based on plasmid DNA (pDNA) were extensively evaluated in vitro as potential treatments of inherited diseases [1]. However, their failure to demonstrate potency at a clinical level due to their inability to bypass hurdles posed by the nuclear membrane of non-dividing cells and immunogenic responses of cytosine-phosphate-guanine (CpG) motifs contained by unmethylated DNA has aroused interest in using mRNA instead of pDNA [2,3].

Since an early study conducted by Malone and co-workers, the use of mRNA in gene therapy was limited by the belief that mRNA is too unstable when transfected into cells [4,5]. Recently, researchers have disproved that notion by successfully demonstrating the feasibility of mRNA-based modalities in several therapeutic applications, including tumor vaccination [6] and cancer immunotherapy. The feasibility and non-toxicity of naked mRNA and mRNA complexed with protamine were demonstrated in human patients via intradermal injections, resulting in promising immunological responses [7,8].

The recent interest in mRNA-based systems is due to the pharmaceutical safety advantages demonstrated over their pDNA-based counterparts. These include, first, the ease of mRNA to be formulated into an efficient therapeutic agent since it does not require the incorporation of promoters and terminators such as pDNA. It lacks immunogenic CpG motifs, which are present in pDNA, and does not need to traverse the nuclear membrane to elicit expression, as it is delivered into the cytoplasm, resulting in early and improved transfection activities [9]. Lastly, mRNA can transfect non-dividing cells, and its inability to integrate into the host genome eliminates insertional mutagenesis, making it safer to deliver than pDNA [10]. However, few studies have explored mRNA transfection over the years, and consequently, knowledge regarding mRNA transfection is limited, as the application of mRNA is still restricted by the need for improved delivery systems [11]. Thus far, the general consensus is that the use of cationic non-viral mRNA-based delivery systems, particularly cationic polymers (e.g., dendrimers), results in significantly improved transgene activity compared to that elicited by pDNA-based delivery systems [5], with some researchers recently using lipid nanoparticles (LNPs) for mRNA delivery [12]. Dendrimers, particularly PAMAM, are shown to elicit high transfection activities in vitro due to their hyperbranched, well-defined, three-dimensional (3D) structure with multiple surface functionalities, extreme buffering capacity, and ability to be protonated at physiological pH for efficient nucleic acid binding [13,14,15,16]. However, their high cytotoxic profiles induced by an excess of the surface amines (tertiary, 3° internal and peripheral primary, 1°) amines, especially at higher generations (>5), have tarnished their use in drug/gene delivery in the past [17]. Many reports, however, have shown that modifying these surface amines via pegylation, methylation, alkylation, acetylation, and conjugation with vitamins or amino acids significantly reduced this cytotoxicity [18,19,20].

Recently, several studies have exploited the remarkable properties of dendrimers as stabilizers of metal nanoparticles (NPs) [14,15,16,21,22,23]. This strategy combines the unique properties of metal NPs with those of cationic dendrimers to produce safe and highly efficient non-viral gene delivery systems. Gold nanoparticles are among the most commonly used metallic NPs to date due to their facile synthesis, biocompatibility, favorable surface-to-volume ratio, ability to be modified, and low cytotoxicity [24,25]. To the best of our knowledge, the transfection of mRNA using PAMAM dendrimer-grafted gold nanoparticles (AuNPs) was never explored. For that reason, this proof of principle study focused on designing FA-modified PAMAM-grafted AuNPs and PAMAM-grafted AuNPs and evaluating their cytotoxicity profiles and capacity to efficiently deliver F*Luc*-mRNA in vitro. FA-modified PAMAM nano-conjugates and PAMAM nano-conjugates were also evaluated for comparison purposes.

## 2. Materials and Methods

### 2.1. Materials

Starburst PAMAM dendrimer, generation five (PAMAM G5D), (*Mw* of 28,826, 128 surface amino groups), bicinchoninic acid (BCA), folic acid, 1-(3-dimethylaminopropyl)-3-ethyl carbodiimide (EDC), dimethylformamide (DMF), sodium dodecyl sulfate (SDS), dialysis tubing (*MWCO*, 12,000 Daltons), and ribonuclease A (RNase A) were supplied by Sigma-Aldrich (St. Louis, MO, USA). Ultra-pure DNA-grade agarose was acquired from Bio-Rad Laboratories (Richmond, VA, USA). Tris (hydroxymethyl)-aminomethane hydrochloride (Tris-HCl), 3-(4,5-dimethylthiazol-2-yl)-2,5-diphenyltetrazolium bromide (MTT), 2-[4-(2-hydroxyethyl)-1-piperazinyl] ethane sulphonic acid (HEPES), Dimethyl sulphoxide (DMSO), ethidium bromide (ETB), and gold (III) chloride trihydrate 99% (HAuCl4) were purchased from Merck (Darmstadt, Germany). F*Luc*-mRNA (5-methylcytidine and pseudouridine modified) was purchased from TriLink BioTechnologies, Inc (San Diego, CA, USA). Minimum essential medium (EMEM) containing Earle’s salts and L-glutamine, penicillin (500 units/mL)/streptomycin (5000 µg/mL), and trypsin-versene were purchased from Lonza-BioWhittaker (Walkersville, MD, USA). Fetal bovine serum (FBS) was purchased from Highveld Biological (Lyndhurst, South Africa). Human embryonic kidney (HEK293), hepatocellular carcinoma (HepG2), breast adenocarcinoma (MCF-7), cervical adenocarcinoma cells (KB), and colorectal adenocarcinoma (Caco-2) cells were originally obtained from the American Type Culture Collection (Manassas, VA, USA).

### 2.2. Synthesis of Gold Nanoparticles (AuNPs)

An adaptation of the Turkevich method was followed to synthesize the AuNPs [26]. Briefly, HAuCl_4_ (0.03 M, 0.1 mL) was dissolved in 25 mL of 18 MOhm water, stirred vigorously, and heated for 15 min until boiling. This was followed by the slow addition of 1 mL of 1% trisodium citrate (Na_3_C_6_H_5_O_7_) with stirring until a red color change was produced. The mixture was then removed from the heat and stirred until it cooled to room temperature.

### 2.3. Modification of PAMAM G5D with Folic Acid (FA)

PAMAM G5D (dried under nitrogen) was dissolved in 18 MOhm water and conjugated to folic acid (FA) via carbodiimide chemistry as described previously by the authors [15,16]. FA (2.8 µmol in 3 mL of DMF) was reacted with 38.2 µmol EDC for 45 min with constant stirring under nitrogen. The activated FA was then added slowly with stirring into the dendrimer (3 µmol, 100 µL) solution, and the pH maintained at 9.5. The solution was stirred for 3 days under nitrogen, followed by the removal of unreacted by-products by dialysis (*MWCO* 12 000 Da) against 18 MOhm water for 24 h.

### 2.4. Formulation of Dendrimer-Coated AuNPs (Au:G5D NPs, and Folic-Acid-Targeted, Dendrimer-Coated AuNPs (Au:G5D:FA NPs)

The G5D and previously synthesized G5D:FA (Section 2.3) were conjugated to the citrate-reduced AuNP solution as previously described by the authors [15,16] to produce Au:G5D and Au:G5D:FA NPs in a 25:1 gold/dendrimer molar ratio. NPs were dialyzed as in Section 2.3.

### 2.5. Ultra-Violet (UV) and Proton Nuclear Magnetic Resonance (^1^H NMR) Spectroscopy

Successful functionalization of the G5D and AuNPs was monitored by UV-vis spectroscopy (UV-1650PC, Shimadzu, Japan) using a wavelength range of 200–800 nm. Further confirmation of NP synthesis was achieved using ^1^H NMR spectroscopy (Bruker DRX 400) with deuterated (D_2_O) water as a solvent.

### 2.6. Transmission Electron Microscopy (TEM) and Nanoparticle Tracking Analysis (NTA)

The ultrastructural morphology of the NPs and their mRNA nanocomplexes at optimum binding ratios (*w*/*w*) were determined by cryo-TEM, using a Jeol JEM-1010 transmission electron microscope containing a Soft Imaging System (SIS) fitted with a MegaView III digital camera with iTEM UIP software, operating at an acceleration voltage of 200 kV (Tokyo, Japan). The z-average hydrodynamic diameters and zeta (ζ) potentials were determined by nanoparticle tracking analysis (NTA, NanoSight NS500; Malvern Instruments, Worcestershire, UK) at 25 °C. NPs (1 mL) were diluted 1:100 in 18 MOhm and sonicated before analysis. Although the characterization of these NPs was reported previously by the authors [15,16], the mRNA-based nanocomplexes are reported here for the first time.

### 2.7. Nanocomplex Preparation and Binding Studies

Nanocomplexes for mRNA binding, cell viability, and transfection studies contained a constant amount of F*Luc*-mRNA (0.05 µg) together with increasing amounts of G5D, Au:G5D, G5D:FA, and Au:G5D:FA NPs. Nanocomplexes were briefly mixed and incubated at room temperature for 60 min.

#### 2.7.1. Band Shift Assay

Band shift assays [27] were utilized to determine the binding of mRNA to the NPs. Nanocomplexes prepared as in Section 2.7 were subjected to electrophoresis on 1% (*w*/*v*) agarose gels containing ethidium bromide (ETB) (1 μg/mL) in a Bio-Rad mini-sub electrophoresis apparatus containing 1× electrophoresis buffer (36 mM Tris-HCl, 30 mM, sodium phosphate (NaH_2_PO_4_), 10 mM ethylenediamine tetra-acetic acid (EDTA), pH 7.5), for 45 min at 50 Volts. Gels were viewed and images captured using a Vacutec Syngene G: Box BioImaging system (Syngene, Cambridge, UK).

#### 2.7.2. Ethidium Bromide Displacement Assay

The compaction of the nanocomplexes was assessed using a dye displacement assay [27]. ETB solution (24 µL, 100 µg/mL) and HBS (100 µL) were initially added to a 96-well FluorTrac flat-bottom black plate, and fluorescence read in a Glomax^®^-Multi + detection system (Promega, Sunnyvale, CA, USA) at an excitation wavelength of 520 nm and an emission wavelength of 600 nm. This measurement was set as 0% relative fluorescence (RF). The 100% RF was obtained after the addition of 0.05 µg F*Luc*-mRNA. Thereafter, 1 µL aliquots of the respective NPs were added, and fluorescence was measured until a plateau in fluorescence was achieved.

#### 2.7.3. RNase A Protection Assay

The stability of the nanocomplexes and the protection afforded to the mRNA in the presence of degrading enzymes were evaluated by an RNase protection assay adapted from [27]. NP:mRNA nanocomplexes prepared at the sub-optimum, optimum, and supra-optimum ratios (obtained from Section 2.7.1) were exposed to 10% RNase A for 2 h at 37 °C. This was followed by the addition of 10 mM EDTA to halt the reaction and 0.5% SDS to release the nucleic acid from the nanocomplex. Samples were subsequently incubated at 55 °C for 20 min, followed by electrophoresis as described previously (Section 2.7.1).

### 2.8. Cell Culture-Based Assays

All cells were maintained and propagated at 37 °C and 5% CO_2_ in 25 cm^2^ flasks containing sterile EMEM, FBS (10%, *v*/*v*), penicillin G (100 U/mL), and streptomycin sulfate (100 μg/mL). The cells were split upon confluency into desired ratios when necessary and the medium changed routinely.

#### 2.8.1. MTT Cell Viability Assay

The MTT assay was used to determine the viability of the cells after treatment with the respective nanocomplexes as described previously [28,29]. All cells were seeded into 48-well plates at densities of 2.5 × 10^5^ cells/well, and incubated for 24 h at 37 °C. Thereafter, nanocomplexes at selected ratios were added in triplicate, and cells were incubated for 48 h at 37 °C. Cells containing no nanocomplexes were used as the positive control (100% cell viability). Following the 48 h incubation, a fresh medium containing the 10% MTT reagent (5 mg/mL in PBS) was added, followed by a 4 h incubation at 37 °C. The medium MTT mixture was then aspirated, cells washed with PBS (2 × 0.3 mL), and 0.3 mL of DMSO was added to solubilize the resulting formazan crystals. Absorbance was then measured at 570 nm in a Mindray MR-96A microplate reader (Vacutec, Hamburg, Germany) using DMSO as the blank. The percentage cell viability was calculated against the positive control (100%).

#### 2.8.2. Apoptosis Assay

To determine if apoptosis was instrumental in the cell death recorded, a fluorescent dual-stain apoptosis assay was conducted as previously described [30]. Cells (2.9 × 10^5^ cells/well) were plated into 12-well plates and incubated for 24 h at 37 °C. Following the addition of nanocomplexes at optimum binding ratios, the cells were incubated for 48 h at 37 °C. Thereafter, cells were washed with PBS, and 10 μL of AO/ETB (AO/ETB, 1:1 *v*/*v*, 100 μg/mL) was added. Cells were viewed for structural and morphological changes under an Olympus fluorescent microscope (×200 magnification), fitted with a CC12 fluorescent camera (Olympus Co., Tokyo, Japan). Apoptosis was quantified by calculating the apoptotic index (AI) as below:

Apoptotic Index = Number of apoptotic cells/Total number of cells

#### 2.8.3. Transfection and Competition Assays

The transfection and competition assays were conducted as previously described [15,16,28,29]. Cells with densities of 2.5 × 10^5^ cells/well were seeded into 48-well plates and incubated for 24 h at 37 °C. The nanocomplexes (ratios as used for the MTT assay) were then added, and the cells were incubated for 48 h at 37 °C. Thereafter, the cells were washed with PBS (2 × 0.5 mL) and lysed with 80 μL/well cell lysis buffer (Promega) for 15 min with shaking at 30 rpm in a Scientific STR 6 platform rocker (Stuart Scientific, Staffordshire, UK). Cell suspensions were then centrifuged at 12,000× *g* for 1 min. The cell-free extract (20 μL) was added to 100 μL luciferase assay reagent, mixed, and luminescence recorded in relative light units (RLU) in a Glomax^®^-Multi+Detection System (Promega Biosystem, Sunnyvale, CA, USA). The standard BCA assay was used to determine the protein concentrations of the cell-free extracts as described previously [29,31]. The luminescence recorded was normalized against the protein concentration, and luciferase activity was expressed as RLU/mg protein.

For the competition assay, cells were seeded and treated as for the normal transfection, but FA (250 µg) was incubated with folate receptor-positive cells (MCF-7 and KB cells) for 20 min at 37 °C before the addition of the targeted nanocomplexes. Luciferase activity was then determined as described above.

### 2.9. Statistical Analysis

Cell viability and transfection studies were performed in triplicate and results expressed as means ± standard deviation (SD). The experimental data was analyzed by a two-way ANOVA and *t*-test using GraphPad Prism 6.0 and statistically significant values are indicated by * *p* < 0.05, ** *p* < 0.01, *** *p* < 0.001, and **** *p* < 0.0001, # *p* > 0.05.

## 3. Results

### 3.1. UV-Visible and ^1^H NMR Spectroscopy

**Figure 1:**
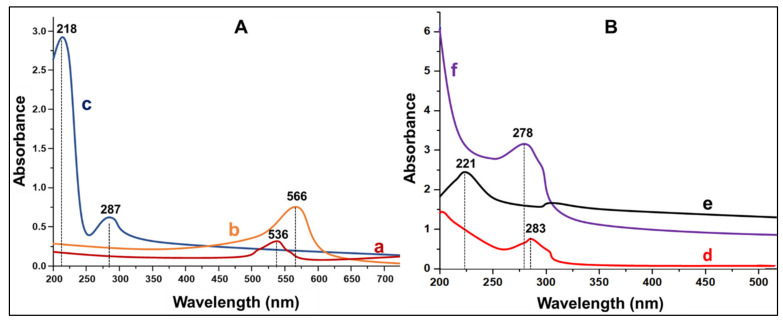
(**A**) UV spectra of (**a**) AuNPs, (**b**) Au:G5D NPs, (**c**) Au:G5D:FA NPs; and (**B**) UV-spectra of (**d**) G5D, (**e**) G5D:FA NPs, and (**f**) FA.

The attachment of G5D and FA on the AuNPs was first confirmed by UV-vis spectroscopy (Figure 1).

The absorption band at 536 nm confirmed the formation of AuNPs, since the known absorption band of AuNPs range between 520 and 550 nm [32]. The band shift from 536 nm to 566 nm confirmed the attachment of G5D on the surface of the AuNPs [33]. Furthermore, the covalent attachment of the FA onto the surface of NPs is known by its absorption maxima at 280 nm with a saddle point at 360 nm [34,35] (Figure 1A), corresponding to the absorption peak of Au:G5D:FA observed at 287 nm. Figure 1B shows the λ max for G5D and FA which caused the changes in the surface plasmon resonance of the AuNPs upon functionalization. The UV-vis absorbances were further utilized to estimate the amount of bound G5D and FA, which were 53.8% and 60.6%, respectively.

**Figure 2:**
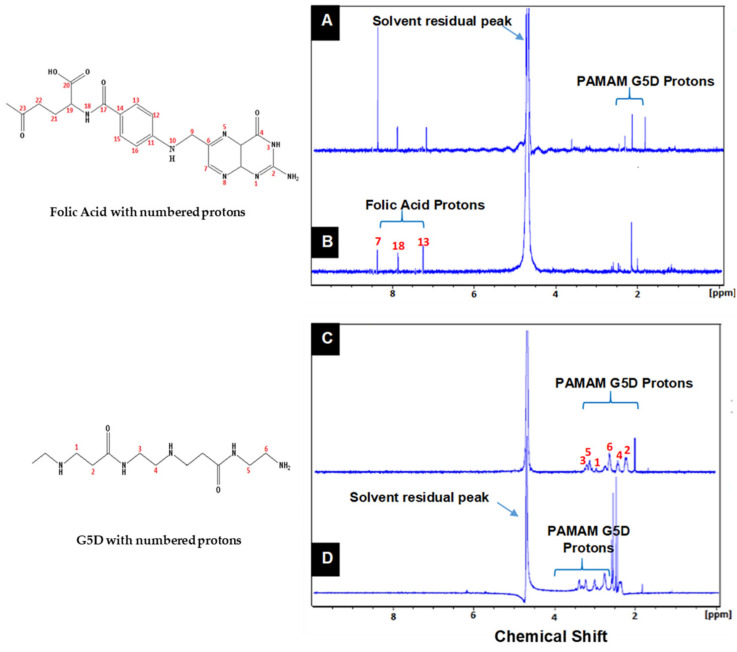
The ^1^H NMR spectra of PAMAM dendrimer (G5D) and folic acid-functionalized gold nanoparticles in D_2_O. (**A**) G5D:FA, (**B**) Au:G5D:FA, (**C**) G5D, (**D**) Au:G5D.

The formation of Au:G5D and Au:G5D:FA NPs was also verified by ^1^H NMR spectroscopy (Figure 2). Significant differences in the chemical shift of protons related to Au:G5D (D), Au:G5D:FA (B), G5D:FA (A) were observed when compared to G5D(C). The ^1^H NMR of the G5D shows six broad peaks (Figure 2C, peaks 1–6) as indicated by a chemical shift ranging from 2.25 to 3.34 ppm, representing the protons of the amino (NH_2_) and methylene groups (CH_2_). These findings correlated with those reported [36,37]. Moreover, the three peaks between 6.50 and 8.63 ppm observed in Figure 2A,B indicate the attachment of FA protons (H-Ar (7 and 13), NH (18)). The formation of Au:G5D nanocomplexes resulted in the downfield shift of protons 4, 5, and 6 of G5D, which indicated the interaction of the surface of the AuNPs with the internal amines of the dendrimer. These findings correlate to that in literature [38].

### 3.2. Morphology, Size, and Zeta Potential of Nanoparticles and Nanocomplexes

**Figure 3:**
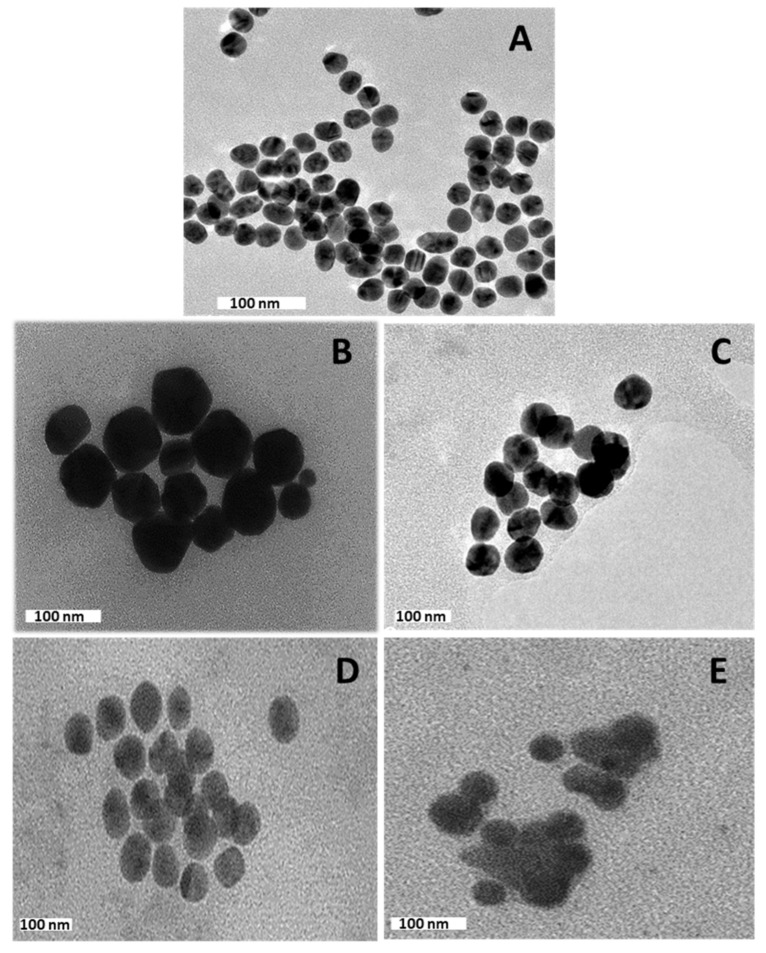
TEM micrograph of (**A**) AuNPs, (**B**) Au:G5D, (**C**) Au:G5D-mRNA nanocomplex, (**D**) Au:G5D:FA, and (**E**) Au:G5D:FA-mRNA nanocomplex. Nanocomplexes were prepared at optimum binding ratios of 3:1 (*w*/*w*) for Au:G5D-mRNA and 4:1 (*w*/*w*) for Au:G5D:FA-mRNA, respectively.

The NPs appeared spherical (Figure 3A,B,D) with a uniform distribution and hydrodynamic diameters from NTA ranging from 65 nm to 128 nm (Table 1). Nanocomplexes prepared at optimum binding ratios (Figure 3C,E), presented as clusters of smaller particles with hydrodynamic diameters ranging from 101 nm to 265 nm (Table 1). There was no significant size difference (# *p* > 0.05) between the Au:G5D/Au:G5D:FA and G5D/G5D:FA nanocomplexes (Table 1).

Overall, ζ potentials ranged from 20.9 mV to 87.2 mV for the NPs and from −21.0 mV to −65 mV for the nanocomplexes, indicating good colloidal stability (Table 1). Au:G5D and Au:G5D:FA nanocomplexes had the highest ζ potentials of −37.3 mV and −65.7 mV, respectively. The polydispersity indices (PDI) revealed that all the NPs and nanocomplexes are highly monodisperse and uniform in size with PDI values below 0.2 (Table 1), suggesting that these NPs and nanocomplexes have a lower tendency to agglomerate [39].

### 3.3. The Band Shift Assay

**Figure 4:**
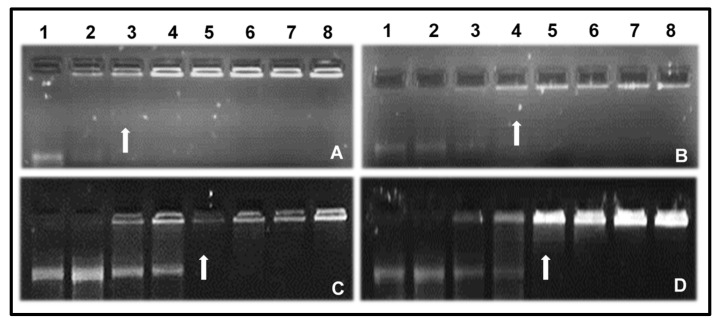
Band shift assay of the interaction between (**A**) G5D, (**B**) Au:G5D, (**C**) G5D:FA, (**D**) Au:G5D:FA, and mRNA. Incubation mixtures (20 µL) in HBS contained varying amounts of the nanoparticle preparation and 0.05 µg FLuc-mRNA corresponding to *w*/*w* ratios of 1:1, 2:1, 3:1, 4:1, 5:1, 6:1, 7:1, and 8:1 in lanes 2–8, respectively (**A**–**D**). Lane 1: naked mRNA control. Arrows indicate endpoint ratios.

The binding of mRNA to the prepared NPs can be seen in Figure 4.

All prepared NPs were able to bind and complex with the mRNA. This can be credited to the ability of G5D to become protonated at physiological pH [11]. G5D and Au:G5D NPs completely retarded the mRNA at ratios of 2:1 and 3:1 (*w*/*w*), respectively, while both G5D:FA and Au:G5D:FA NPs completely retarded mRNA at a ratio of 4:1 (*w*/*w*).

### 3.4. Ethidium Bromide Dye Displacement Assay

**Figure 5:**
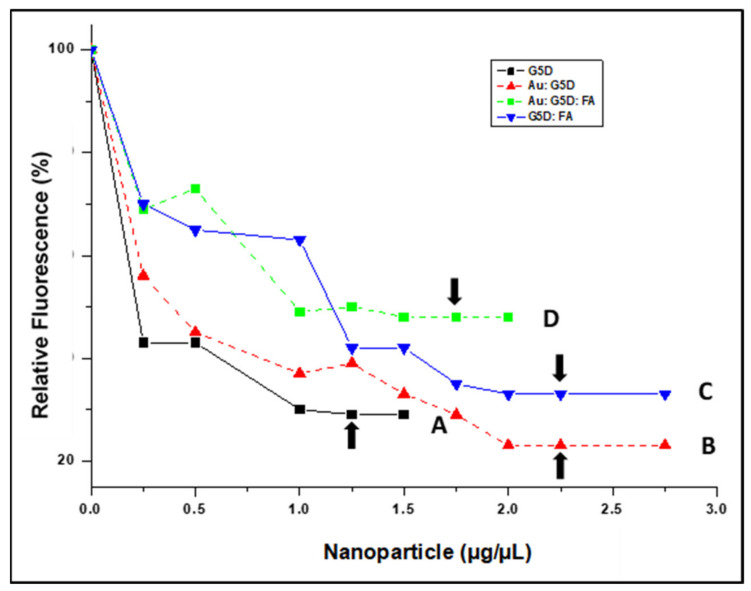
Ethidium bromide displacement assay of (**A**) G5D, (**B**) Au:G5D, (**C**) G5D:FA, and (**D**) Au:G5D:FA NPs. Arrows indicate a point of complexation.

All NPs displaced ethidium bromide (ETB), indicating a significant degree of mRNA compaction, which bodes well for their stability and protection under physiological conditions. The degree of mRNA compaction by the G5D and Au:G5D NPs ranged from 50 to 80%, while that of G5D:FA and Au:G5D:FA NPs ranged from 40 to 70% (Figure 5).

### 3.5. RNase A Digestion Assay

To assess the ability of the NPs to protect the mRNA cargo against nucleases, which would be encountered in circulation in an in vivo system, an RNase A digestion assay was conducted.

**Figure 6:**
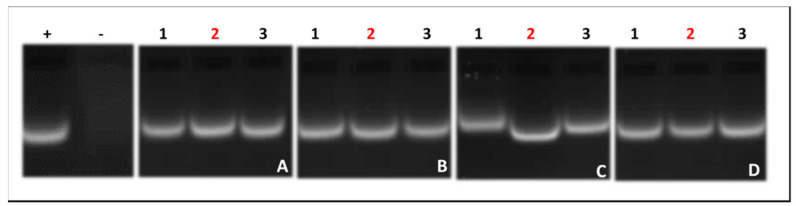
RNase A digestion assay of nanocomplexes. (**A**) G5D, (**B**) Au:G5D, (**C**) G5D: FA, (**D**) Au:G5D:FA. Control: naked mRNA in the absence (+ = positive control) or presence (− = negative control) of RNase A. Lanes 1–3 contain nanocomplexes at sub-optimum, optimum, and supra-optimum nanoparticle: mRNA ratios. (**A**) 1:1, 2:1, 3:1; (**B**) 2:1, 3:1, 4:1; (**C**) 3:1, 4:1, 5:1; (**D**) 3:1, 4:1, 5:1 (*w*/*w*). Red-colored numbers indicate the optimum binding ratios.

Figure 6 clearly shows the exceptional ability of all NPs to fully protect mRNA following treatment with 10% RNase A, as depicted by the presence of undigested bands in all tested ratios. By contrast, the treatment of naked mRNA with RNase A showed complete degradation (negative control), as illustrated in Lane 2.

### 3.6. The MTT Assay

**Figure 7:**
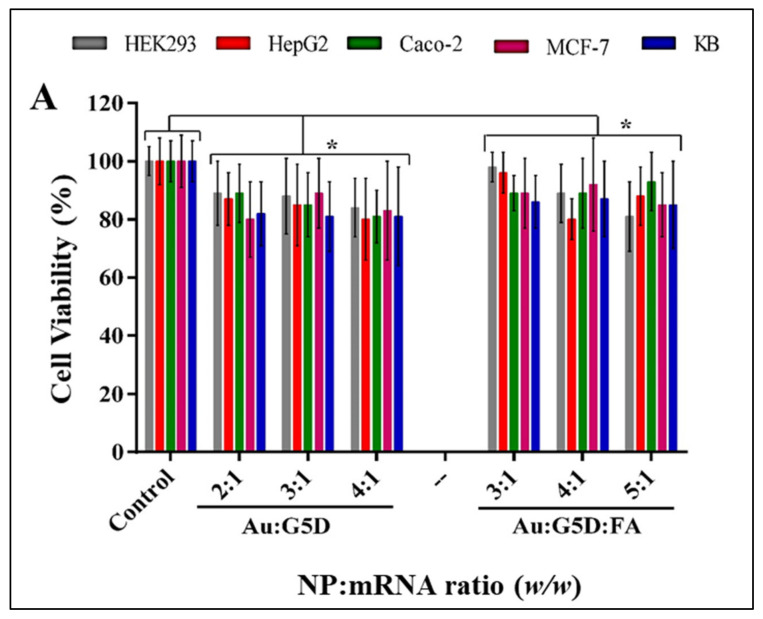

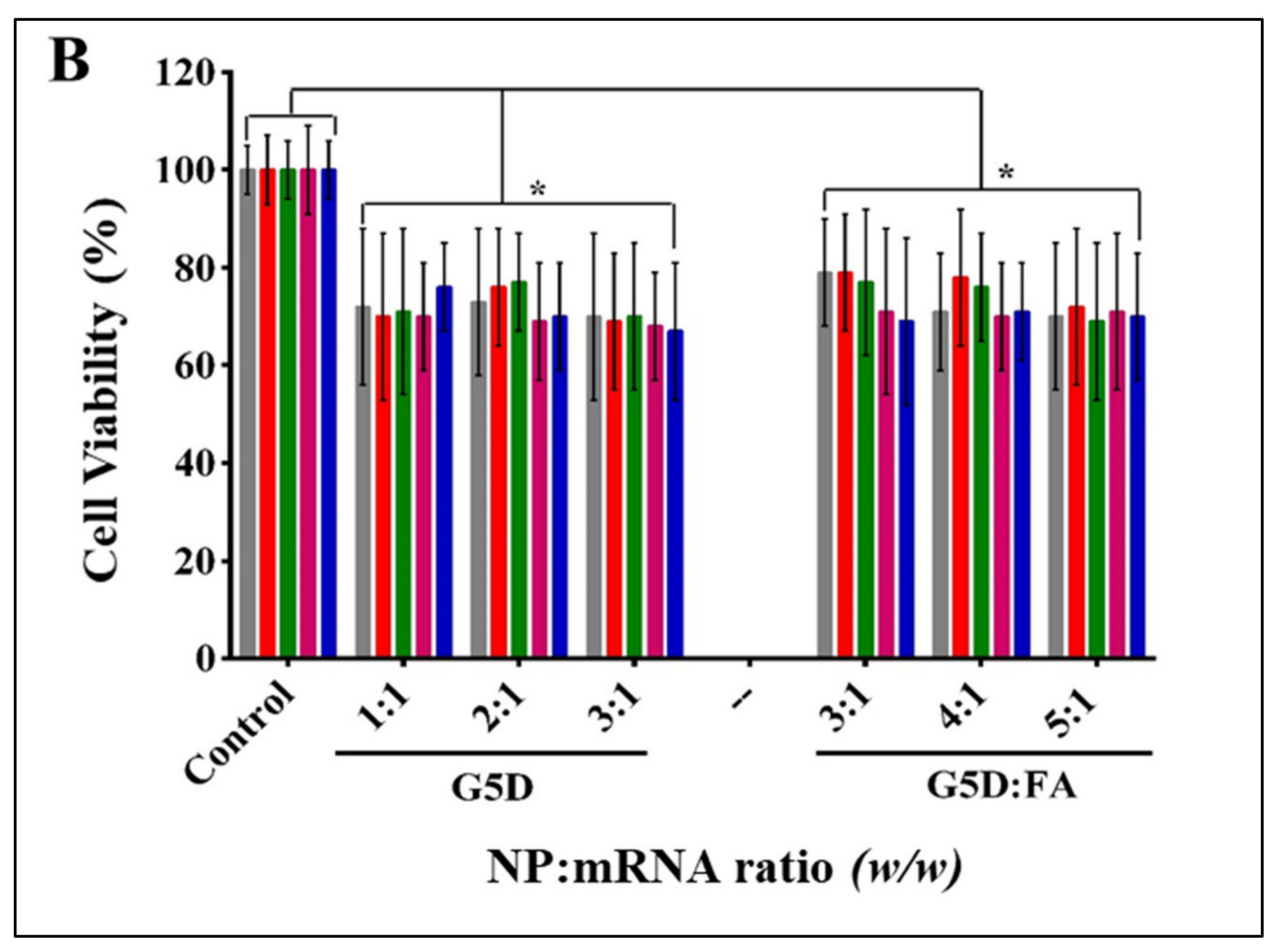
Cell viability assay of nanocomplexes containing (**A**) Au:G5D and Au:G5D:FA; and (**B**) G5D and G5D:FA, in HEK293, HepG2, Caco-2, MCF-7, and KB cells. Cells were incubated with nanocomplexes containing 0.05 μg F*Luc*-mRNA at indicated ratios (*w*/*w*). Nanocomplexes were prepared at sub-optimum, optimum, and supra-optimum ratios. Data are presented as means ± SD (*n* = 3). Control = untreated cells. * *p* > 0.05.

To monitor cell viability after treatment with prepared nanocomplexes in selected cell lines, the MTT assay was conducted. This assay uses the MTT reagent, which enters the cells and passes into the mitochondria, where it is reduced to an insoluble, purple-colored formazan product that can be quantified spectroscopically and used as an indication of metabolically active cells. No significant (*p* > 0.05) change in cell viability was observed following treatment with all nanocomplexes. Higher cell viabilities (80–97%) were observed in all cell lines for the Au:G5D:mRNA and Au:G5D:FA:mRNA nanocomplexes, compared to the G5D:mRNA and G5D:FA:mRNA nanocomplexes (68–78%) (Figure 7A,B).

Noticeably, all FA-targeted nanocomplexes showed higher cell viability than their untargeted nanocomplex counterparts (average cell viability of 88% for Au:G5D:FA and 72% for G5D:FA).

### 3.7. Apoptosis Assay

**Figure 8:**
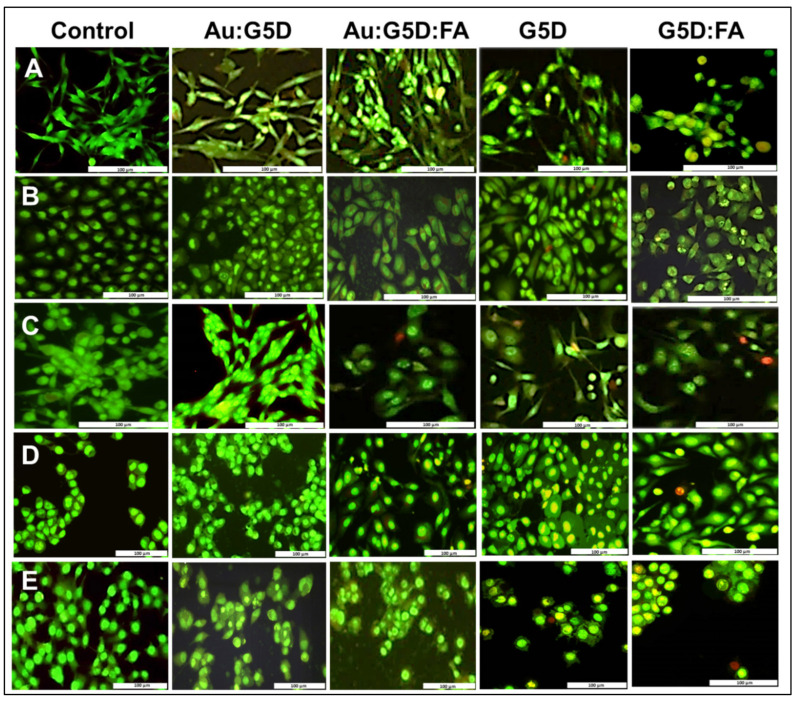
Fluorescence images of (**A**) HEK293, (**B**) HepG2, (C) Caco-2, (**D**) MCF-7, and (**E**) KB cells treated with test and control nanocomplexes prepared at sub-optimum ratios for 24 h, showing induction of apoptosis. Green = live (L), light orange = early apoptotic (EA), and dark orange = late apoptotic (LA) cells. Scale = 100 μm.

Cell death was also studied by evaluating the ability of NPs to induce apoptosis in selected cell lines. All nanocomplexes induced little or no apoptosis in the cells, as evidenced by very few apoptotic (yellow-orange/red) cells visible and low apoptotic indices (AI) (Figure 8 and Table 2). Noticeably, the AI values of Au:G5D:mRNA and Au:G5D:FA:mRNA nanocomplexes were significantly (*p* < 0.0001) lower than those of the G5D:mRNA and G5D:FA:mRNA nanocomplexes particularly, in all cell lines (Table 2).

### 3.8. Transfection and Competition Assays

**Figure 9:**
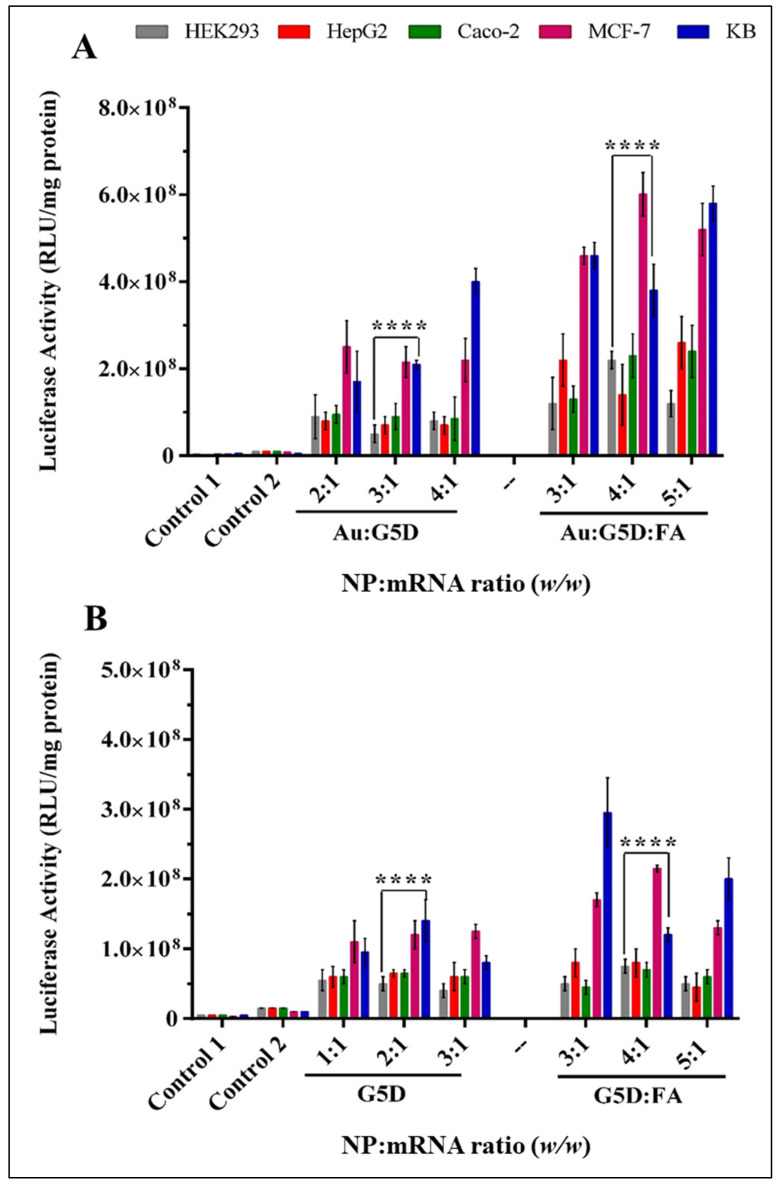
Transgene expression for (**A**) Au:G5D and Au:G5D:FA nanocomplexes, and (**B**) G5D and G5D:FA nanocomplexes in HEK293, HepG2, Caco-2, MCF-7, and KB cells. Nanocomplexes contained 0.05 µg mRNA with varying amounts of nanoparticles to constitute the sub-optimum, optimum, and supra-optimum (*w*/*w*) ratios. Control 1 = untreated cells. Control 2 = cells treated with naked F*Luc*-mRNA. The transgene expression is reported as RLU/mg protein. Data are presented as means ± SD (*n* = 3). **** *p* < 0.0001 for optimum ratios.

The ability of the NPs to deliver mRNA was evaluated in folate receptor-negative cell lines, HEK293, Caco-2, and folate receptor-positive cell lines HepG2, MCF-7, and KB (KB > MCF-7 > HepG2), with KB cells often being used as a model for folate receptors (FRs) [40]. The transfection efficacy of the nanocomplexes was assessed as a function of weight ratios (sub-optimum, optimum, and supra-optimum). The transfection activity of the Au:G5D:mRNA and Au:G5D:FA:mRNA nanocomplexes (Figure 9A,B) was much higher than that of the naked mRNA (control). Moreover, the transfection levels in HEK293 and Caco-2 cells were significantly (*p* < 0.001) lower than those elicited in the receptor-positive cells.

All nanocomplexes showed excellent transfection activity, with Au:G5D:mRNA and Au:G5D:FA:mRNA nanocomplexes (Figure 9A) showing higher transfection efficiencies ranging from 5 × 10^7^–6 × 10^8^ RLU/mg protein. On the other hand, G5D:mRNA and G5D:FA:mRNA nanocomplexes (4 × 10^7^–3 × 10^8^ RLU/mg protein) produced decreased transfection activity (Figure 9B). Noticeably, the Au:G5D:FA:mRNA nanocomplexes showed a four-fold increase in transfection activity (6 × 10^8^ RLU/mg protein), compared to Au:G5D:mRNA nanocomplexes (2 × 10^8^ RLU/mg protein) at the optimum ratios in the FR positive cell line, MCF-7.

**Figure 10:**
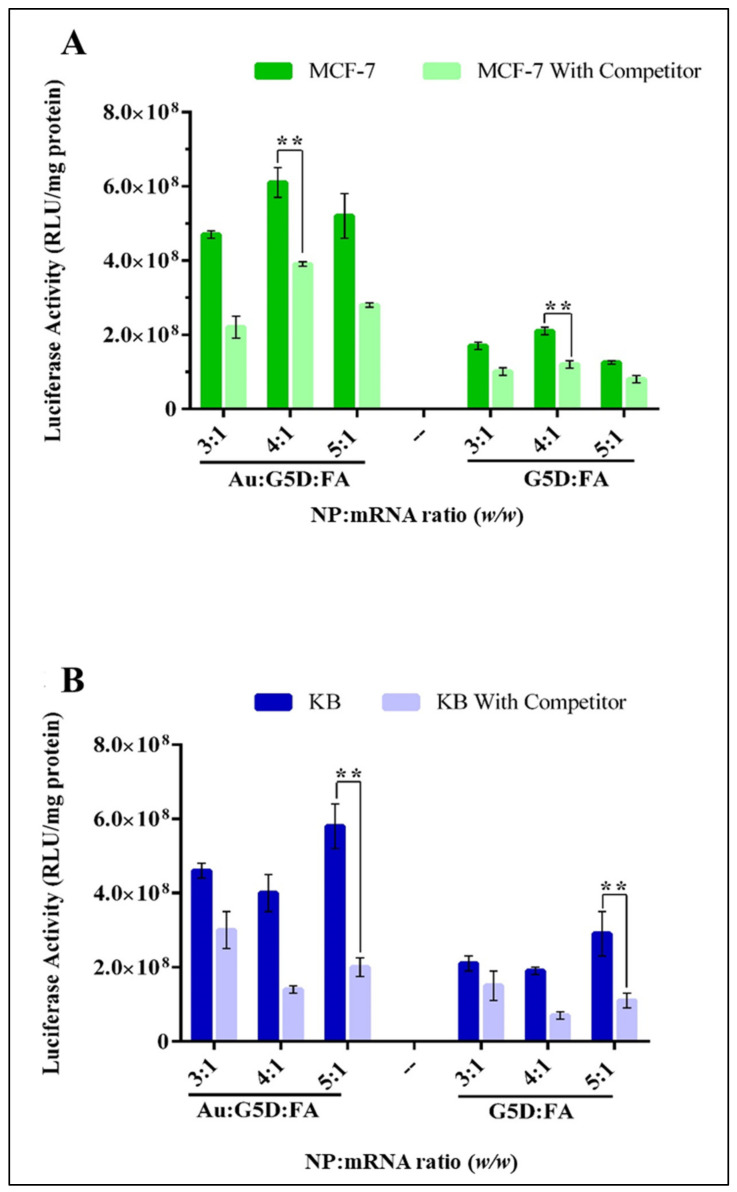
Competition studies of FA-targeted mRNA nanocomplexes in (**A**) MCF-7 and (**B**) KB cells. Cells were first exposed to excess folic acid (250 μg) then treated with FA-targeted nanocomplexes at selected ratios. Transgene expression is reported as RLU/mg protein. Data are presented as means ± SD (*n* = 3). ** *p* < 0.01.

To confirm the mechanism of the cellular uptake of the nanocomplexes, a competition assay was conducted. This involved flooding the cells with excess free FA (250 μg) before exposure to the FA-targeted nanocomplexes (Au:G5D:FA:mRNA and G5D:FA:mRNA). The assay was conducted in the cell lines with overall higher targeted transgene expression, viz. MCF-7 and KB cell. A significant (*p* < 0.01) drop of approximately 30% in transgene activity was observed as depicted in Figure 10, which suggests that a large portion of these nanocomplexes were taken up by receptor-mediated endocytosis [41], confirming that FA receptor mediation was a key player in the high transgene expression obtained.

## 4. Discussion

All NPs were successfully synthesized to produce spherical, monodispersed NPs. NP synthesis was confirmed by UV-vis and NMR spectroscopy, which also confirmed that the G5D polymer and FA moiety were successfully conjugated to the AuNPs. The Au:G5D NPs produced a redshift in the spectrum, whereas the Au:G5D:FA NPs produced a blue shift. The G5D generally has a very weak peak between 280 and 285 nm [14], especially at higher or at physiological pH due to the presence of the protonated amine groups of the G5D [14,42]. In this study, a small peak was noted at 283 nm. However, this peak often seems to disappear at lower pH. In NMR, the formation of Au:G5D NPs resulted in the downfield shift of protons 4, 5, and 6 of G5D, which indicated the interaction of the surface of the AuNPs with the internal amines of the dendrimers [43].

Favorably sized NPs (<200 nm) with the most zeta potentials, except for the AuNPs on their own being above 20 mV, were produced. All nanocomplexes, with the exception of the G5D:FA nanocomplexes (265.2 nm) fell within the ideal size range (100–200 nm) required for gene delivery via non-specific or receptor-specific uptake [44,45,46]. Zeta potential measurements greater than +25 mV or less than −25 mV are reported to be associated with good colloidal stability [47]. These AuNPs alone showed poor stability (−7.3 mV), but upon G5D and FA functionalization, the stability improved immensely to +20.9 mV for Au:G5D and +29 mV for Au:G5D:FA. This confirms that the functionalization of the NPs with the dendrimers improved their stability, as seen in a recent study where dendrimer was used to functionalize selenium NPs [14]. The improved stability achieved with the targeted NPs could be due to the partial shielding effect imparted by FA, in addition to the repulsive cationic amine groups on the dendrimers, which prevents particle aggregation [14,48]. From these findings, it can be predicted that these nanocomplexes may be efficient in delivering mRNA.

The differences observed in the binding efficiency between the FA-targeted and untargeted NPs could be due to the possible shielding of the cationic charges of the dendrimers on the targeted NPs by the FA moiety, which meant that more positive charges and more NPs were required to fully neutralize the negative charges on the mRNA [49]. Overall, the NP:mRNA nanocomplex formation occurred at very low ratios (*w*/*w*), which could be accredited to the single-stranded nature of the mRNA, which is quickly embedded by the highly cationic G5D. The G5D and Au:G5D showed greater quenching of the ethidium bromide fluorescence, which could be attributed to more amine groups being available to bind the mRNA [14]. The compaction was seen for the targeted nanocomplexes, further suggested a weaker binding of the mRNA, which could translate into easy dissociation of the mRNA from the nanocomplexes during transfection, hence avoiding degradation by the lysosomal compartment, and in turn, enhancing gene-transfection efficiency [50]. Overall, all NPs were able to efficiently bind and compact mRNA to varying degrees.

The integrity of the nanocomplexes may be compromised by degrading nuclease enzymes such as RNase A, leading to a reduced transgene expression [51]. The good nuclease protection afforded by the NPs in this study could be due to the highly organized globular structures that formed as a result of the electrostatic interaction between the negatively charged single-stranded mRNA and the highly cationic G5D-containing NPs [52]. The use of the RNase enzyme was a stringent test for these NPs due to its specificity for RNA molecules and was reported previously [53,54]. Various studies have used the less-specific fetal bovine serum containing nucleases to determine the integrity of RNA-based nanocomplexes [55,56] to achieve similar results. In the circulatory system, it is possible that the nanoparticles may encounter less-specific enzymes and possibly at lower concentrations as well. However, this assay confirmed that all NPs afforded exceptional protection to the mRNA cargo, boding well for future in vivo studies.

The first step towards understanding the biocompatibility of a delivery system often involves the use of cell-culture-based studies, commencing with the assessment of cytotoxicity. The gold-containing NPs achieved higher cell viability, which may be due to the presence of the gold in the NP and partly to the reduction of the cationic charges of the 1° amines of the G5D, some of which are responsible for stabilizing the entrapped AuNPs [21]. Furthermore, unmodified AuNPs have been shown to have little or no impact on cytotoxicity in non-cancer HEK293 and cervical cancer (HeLa) cells [24], which could be attributed to their inherent biocompatibility and favorable physicochemical properties that have been widely mentioned. Noticeably, all FA-targeted nanocomplexes showed higher cell viabilities compared to their untargeted nanocomplex counterparts, which could be as a result of the shielding effect of FA, which may have covered a portion of the positive charges on the surface of G5D, hence reducing the strong electrostatic interaction between the cells and the NPs [17]. Overall, more than 80% of cells were still viable after being exposed to the gold-containing nanocomplexes at the selected ratios, suggesting that these nanocomplexes were superior and well-tolerated in all tested cell lines, and therefore relatively safe to use. Apoptosis studies corroborated these results, confirming that the Au:G5D:mRNA and Au:G5D:FA:mRNA nanocomplexes were safe and stable and did not induce any significant apoptotic effects.

The introduction of naked mRNA into cells is known to be associated with poor transgene expression, mainly due to enzymatic degradation [57], as evidenced in the RNase A digestion assay. All nanocomplexes displayed significant transfection in the cell lines tested. The Au:G5D:mRNA and Au:G5D:FA:mRNA nanocomplexes produced the highest luciferase activity, which could be due to three reasons. First, since the translation of mRNA occurred in the cytoplasm—and the major limiting step, which is the nuclear pore entry, was avoided—resulting in an increased transgene expression. Second, the transfection studies were conducted over a duration of 48 h, and more protein may have expressed, considering that mRNA may have a limited half-life [5]. Lastly, the efficient encapsulation of the mRNA by the dendrimer and its exceptional buffering capacity could have helped protect the mRNA from degradation and facilitated the endosomal escape of the nanocomplexes [58].

HepG2 cells exhibited lower luciferase expression, possibly due to fewer receptors on their cell surface compared to MCF-7 and KB cells. The low targeted expression is associated with a lack of specific transcription factors and cell-surface receptors [35]. The higher transfection efficiencies of Au:G5D:mRNA and Au:G5D:FA:mRNA nanocomplexes can be accredited to the entrapment of AuNPs within the 1° amines of the dendrimers, which helped preserve the structural integrity of the dendrimers, allowing for efficient interaction between the dendrimers and the mRNA [21]. This could lead to favorable cellular uptake and high gene expression. The decreased transfection activities of the G5D:mRNA and G5D:FA:mRNA nanocomplexes could be due to their higher cytotoxicity compared to their gold-containing counterparts and the poor dissociation between the mRNA and the G5D due to their strong binding affinity. The mRNA may have been entrapped by a network formed by the branches of the dendrimer. Earlier studies have demonstrated a direct correlation between the binding affinity of the single-stranded mRNA to cationic polymers and transgene expression [59].

The Au:G5D:FA:mRNA nanocomplexes showed a superior transfection activity to the Au:G5D:mRNA nanocomplexes, most likely due to ligand–receptor interaction that occurred between the FA and the FRs abundantly, decorating the surface of the MCF-7 and KB cells [60]. It is generally known that FA has a high affinity for FRs overexpressed by a majority of cancer cells [35], with KB cells generally regarded as models for the folate receptor, as previously mentioned [40]. The significant (*p* < 0.01) drop in transgene activity in the competition assay suggested that a large portion of these nanocomplexes were taken up by receptor-mediated endocytosis, confirming that FA receptor-mediation was a key player in the high transgene expression obtained.

## 5. Conclusions

Both Au:G5D and Au:G5D:FA NPs were highly efficient in F*Luc*-mRNA binding and delivery. They formed stable nanocomplexes and afforded excellent protection to the mRNA against RNases. Furthermore, more than 80% cell viability was observed, suggesting that these nanocomplexes were well tolerated by all cells. This was also demonstrated in their superior transfection efficiency, indicating the significant and synergistic roles played by both the dendrimer and the AuNPs in their formulation. This study further confirmed that folate-receptor-mediated delivery was the main route of entry into the receptor-positive cells, as evidenced by the transfection levels in the FA receptor negative cell lines, being significantly lower than that in FA receptor positive cell lines. Since this proof in principle study has shown potential, future studies would encompass the NP optimization for in vivo delivery using a therapeutic mRNA molecule.

## Figures and Tables

**Table 1:** Hydrodynamic size, ζ potential measurements, and polydispersity indices of nanoparticles and nanocomplexes. Data are presented as mean diameter ± standard deviation (SD) (*n* = 3).

Nanoparticles/Nanocomplexes	NP:mRNA (*w*/*w*) Ratio	Mean Diameter (nm) ± SD	ζ Potential (mV) ± SD	Polydispersity Index
Au [16]	-	65.9 ± 9.8	−7.3 ± 1.6	0.022
G5D [16]	-	161.3 ± 11.9	+87.2 ± 2.4	0.005
Au:G5D [16]	-	100.5 ± 44.1	+20.9 ± 2.2	0.193
G5D:FA [16]	-	128.0 ± 1.20	+71.2 ± 3.4	0.00009
Au:G5D:FA [16]	-	77.7 ± 12.5	+29.0 ± 0.5	0.026
Au:G5D-mRNA	3:1	207.2 ± 35.5 #	−37.3 ± 0.1 ***	0.029
Au:G5D:FA-mRNA	4:1	101.8 ± 36.9 #	−65.7 ± 1.4 ***	0.131
G5D-mRNA	2:1	118.0 ± 6.20 #	−21.0 ± 0.5 ***	0.028
G5D:FA-mRNA	4:1	265.2 ± 51.6 #	−25.8 + 0.0 ***	0.038

# *p* > 0.05, *** *p* < 0.001, when dendrimer-only-based nanocomplexes are compared to gold–dendrimer nanocomplexes.

**Table 2:** Apoptotic indices of nanocomplexes in selected cell lines.

Cell Lines	Apoptotic Indices				
	Cell Control	Nanocomplexes			
		Au:G5D	Au:G5D:FA	G5D	G5D:FA
HEK293	0.0	0.03 ± 0.0001	0.04 ± 0.0004	0.07 ± 0.0010	0.08 ± 0.0020
HepG2	0.0	0.06 ± 0.0015	0.04 ± 0.0018	0.08 ± 0.0012	0.09 ± 0.0011
Caco-2	0.0	0.05 ± 0.0010	0.04 ± 0.0011	0.13 ± 0.0015	0.11 ± 0.0030
MCF-7	0.0	0.04 ± 0.0011	0.06 ± 0.0003	0.25 ± 0.0030	0.23 ± 0.0010
KB	0.0	0.05 ± 0.0021	0.06 ± 0.0003	0.19 ± 0.0015	0.20 ± 0.0012

## Data Availability

The data and contributions presented in the study are included in the article. Further inquiries can be directed to the corresponding author.
